# Supplementary material for: Laminar specificity and coverage of viral-mediated gene expression restricted to GABAergic interneurons and their parvalbumin subclass in marmoset primary visual cortex
Source: eLife. 2024 Sep 19;13:RP97673. doi: 10.7554/eLife.97673 (PMC11412690; doi:10.7554/eLife.97673)
Supplement: Supplementary file 1. [file elife-97673-supp1.docx]

**Supplementary File 1. Animals and Viral Injections**

| **AAV-h56D vectors injection parameters** | | | | | | | | | |
| --- | --- | --- | --- | --- | --- | --- | --- | --- | --- |
| **Case No.** | **Deep Inj.** | | **Middle Inj.** | | **Superficial Inj.** | | **Total Volume**  (nl) | **Viral Titer** (gc/ml) | **Survival Time**  (days) |
| ***Serotype*** | **Depth**  (mm) | **Volume**  (nl) | **Depth**  (mm) | **Volume**  (nl) | **Depth**  (mm) | **Volume**  (nl) |  |  |  |
| MM404RH  ***AAV1*** | 1.5 | 200 | 1.0 | 200 | 0.5 | 200 | 600nL | 1.0E14 | 28 |
| MM404RH  ***AAV7*** | 1.5 | 200 | 1.0 | 200 | 0.5 | 200 | 600nL | 1.0E13 | 28 |
| MM404RH  ***AAV9*** | 1.5 | 200 | 1.0 | 200 | 0.5 | 200 | 600nL | 1.0E13 | 28 |

| **AAV-PHP.eB-S5E2 vector injection parameters** | | | | | | | | | |
| --- | --- | --- | --- | --- | --- | --- | --- | --- | --- |
| **Case No.** | **Deep Inj.** | | **Middle Inj.** | | **Superficial Inj.** | | **Total Volume**  (nl) | **Viral Titer** (gc/ml) | **Survival Time**  (days) |
|  | **Depth**  (mm) | **Volume**  (nl) | **Depth**  (mm) | **Volume**  (nl) | **Depth**  (mm) | **Volume**  (nl) |  |  |  |
| MM417RH | 1.3 | 195 | 0.8 | 195 | 0.6 | 195 | 585 | 8.3E12 | 12 |
| MM423LH | 1.2 | 105 | 0.8 | 105 | 0.4 | 105 | 315 | 8.3E12 | 21 |
| MM430LH | 1.2 | 105 | 0.8 | 105 | 0.4 | 105 | 315 | 8.3E12 | 21 |
| MM430RH | 1.2 | 60 | 0.8 | 60 | 0.4 | 60 | 180 | 8.3E12 | 21 |
| MM430LH | 1.2 | 60 | 0.8 | 60 | 0.4 | 60 | 180 | 8.3E12 | 21 |
| MM430RH | 1.2 | 35 | 0.8 | 35 | 0.4 | 35 | 105 | 8.3E12 | 21 |
| MM430LH | 1.2 | 30 | 0.8 | 30 | 0.4 | 30 | 90 | 8.3E12 | 21 |
|  |  |  |  |  |  |  |  |  |  |
