# Supplementary material for: Laminar specificity and coverage of viral-mediated gene expression restricted to GABAergic interneurons and their parvalbumin subclass in marmoset primary visual cortex
Source: eLife. 2024 Sep 19;13:RP97673. doi: 10.7554/eLife.97673 (PMC11412690; doi:10.7554/eLife.97673)
Supplement: Supplementary file 2. [file elife-97673-supp2.docx]

**Supplementary File 2. AAV-PHP.eB-S5E2-tdT specificity and coverage: individual injections**

| **Case No.** | **Tot volume**  (nl) | **Specificity**  (mean±sem%) | **Coverage**  (mean±sem%) | **Total cell count**  (n) |
| --- | --- | --- | --- | --- |
| MM417RH | 585 | 0.79±0.058 | 0.79±0.024 | 283 |
| MM423LH | 315 | 0.76±0.064 | 0.82±0.017 | 313 |
| MM430LH | 315 | 0.89±0.023 | 0.70±0.032 | 459 |
| MM430RH | 180 | 0.95±0.008 | 0.81±0.005 | 369 |
| MM430LH | 180 | 0.94±0.033 | 0.80±0.020 | 298 |
| MM430RH | 105 | 0.97±0.008 | 0.83±0.009 | 276 |
| MM430LH | 90 | 0.80±0.031 | 0.80±0.036 | 280 |
